# Oxidative stress in early childhood caries: evaluation of antioxidant defense and DNA damage biomarkers

**DOI:** 10.1186/s12903-026-09197-3

**Published:** 2026-07-21

**Authors:** Bilge İmrendi Yergin, Volkan Arıkan, Funda Bulut Arıkan

**Affiliations:** 1 Department of Pediatric Dentistry, Faculty of Dentistry, Kirikkale University, Kirikkale, Türkiye; 2 Department of Physiology, Faculty of Medicine, Kirikkale University, Kirikkale, Türkiye

**Keywords:** Early childhood caries, Oxidative stress, Saliva, Total antioxidant capacity, 8-OHdG, DNA damage, Biomarkers

## Abstract

**Background:**

Early childhood caries (ECC) is one of the most prevalent chronic diseases in children and is associated with inflammatory and oxidative processes. This study aimed to evaluate salivary oxidative stress biomarkers and oxidative DNA damage in children with ECC and severe ECC (S-ECC).

**Methods:**

This cross-sectional study included 90 children aged 2–6 years (control, *n* = 29; ECC, *n* = 30; S-ECC, *n* = 31).Salivary total antioxidant status (TAS), total oxidant status (TOS), oxidative stress index (OSI), arylesterase (ARES), asprosin, and 8-hydroxy-2′-deoxyguanosine (8-OHdG) levels were analyzed. Correlation and multivariate regression analyses were performed to evaluate the associations between biomarkers and caries severity.

**Results:**

TAS levels differed significantly among the groups (*p* = 0.014) and were significantly lower in the ECC group than in the control and S-ECC groups. In contrast, 8-OHdG levels were significantly higher in the S-ECC group than in the control group (*p* = 0.013). No significant differences were observed among the groups regarding asprosin (*p* = 0.901), TOS (*p* = 0.865), OSI (*p* = 0.422), or ARES levels (*p* = 0.471). A weak negative correlation was observed between age and 8-OHdG levels (*r* = − 0.253, *p* = 0.016). Multivariable regression analysis demonstrated that ECC was independently associated with lower TAS levels (*p* = 0.011), whereas S-ECC was independently associated with higher 8-OHdG levels (*p* = 0.003).

**Conclusions:**

ECC is associated with alterations in salivary oxidative stress biomarkers, characterized by reduced antioxidant capacity and increased oxidative DNA damage in more severe forms of the disease. These findings suggest that oxidative stress may contribute to ECC progression and support the potential role of salivary biomarkers in evaluating disease severity and oxidative tissue damage during early childhood.

## Introduction

Early childhood caries (ECC) is one of the most common chronic diseases observed during childhood, characterized by rapid progression [1, 2]. ECC not only affects oral health but may also negatively influence children’s quality of life by causing pain, infection, and difficulties in nutrition and speech development [1, 3, 4]. Microbial factors, dietary habits, and host-related factors play important roles in disease development [5].

In recent years, oxidative stress has been suggested to play a significant role in the pathogenesis of ECC. Oxidative stress is defined as an imbalance between increased reactive oxygen species (ROS) production and antioxidant defense mechanisms, which may result in cellular and tissue damage [6, 7]. Salivary total antioxidant capacity (TAS), total oxidant status (TOS), and oxidative stress index (OSI) are widely used biomarkers for evaluating this balance [8]. In addition, 8-hydroxy-2′-deoxyguanosine (8-OHdG) is an important biomarker of oxidative DNA damage [8, 9]. Furthermore, arylesterase (ARES) activity and asprosin levels have recently emerged as potential biomarkers of oxidative stress [10–12]. Experimental studies have suggested that asprosin may participate in oxidative stress and inflammatory pathways by modulating oxidant and antioxidant responses [12]. In addition, increased salivary and serum asprosin levels have been reported in periodontal disease, indicating a potential role in oral inflammatory conditions [13]. Therefore, asprosin may be a promising biomarker for investigating oxidative stress-related mechanisms in ECC.

Although several studies have investigated the relationship between ECC and oxidative stress, the contribution of these biomarkers to disease severity and oxidative DNA damage progression remains unclear. Therefore, this study aimed to evaluate the levels of TAS, TOS, OSI, ARES, asprosin, and 8-OHdG in children with ECC and severe ECC (S-ECC) and to investigate their relationship with caries severity.

## Materials and methods

This study was conducted in 2025 at the Department of Pediatric Dentistry, Faculty of Dentistry, Kırıkkale University, Turkey. A total of 90 children aged 2–6 years participated in this study. The participants were divided into three groups: caries-free control group (*n* = 29), ECC group (*n* = 30), and S-ECC group (*n* = 31).

### Ethical approval

Ethical approval was obtained from the Non-Interventional Research Ethics Committee of Kırıkkale University (Decision No: 2025/04, Date: 26/02/2025). Written informed consent was obtained from the parents of all the participating children before their inclusion in the study.

### Sample size

Sample size calculation was performed using G*Power 3.1.9.6 software based on a one-way analysis of variance (ANOVA) for comparison of the study groups. With an effect size of 0.40, alpha value of 0.05, and statistical power of 95%, the minimum required sample size was determined to be 84 participants. Considering the possible sample loss, a total of 90 children were included in the study.

### Inclusion and exclusion criteria

Children aged 2–6 years with no systemic disease and not using any medications were included in the study. Children with periodontal disease, oral lesions, a history of restorations or tooth extraction, vitamin use, antioxidant supplementation, or cooperation difficulties were excluded from the study.

### Clinical examination and grouping

All clinical examinations were performed by a single, calibrated examiner using a dental mirror and probe under clinical conditions. Participants were classified into caries-free, ECC, and S-ECC groups according to the American Academy of Pediatric Dentistry (AAPD) criteria.

ECC was defined as the presence of one or more decayed (cavitated or non-cavitated), missing due to caries, or filled tooth surfaces in any primary tooth in a child younger than six years of age.

S-ECC was defined as:

1. Any sign of smooth-surface caries in a child younger than 3 years of age
2. One or more cavitated, missing due to caries, or filled smooth surfaces in the primary maxillary anterior teeth in children aged 3–5 years
3. A dmfs score of ≥ 4 at age 3 years, ≥ 5 at age 4 years, or ≥ 6 at age 5 years, according to the AAPD guidelines [14]

### Collection of saliva samples

Unstimulated saliva samples were collected from all participants between 09:00 and 11:00 a.m. following at least 8 h of overnight fasting. To minimize the potential influence of recent food intake and oral hygiene procedures on salivary biomarker levels, the children were instructed not to consume any food or beverages before sample collection and to refrain from tooth brushing and the use of mouth rinses on the morning of sampling. Approximately 2 mL of saliva was collected in sterile tubes using the passive drooling method. The samples were stored at − 20 °C immediately after collection until biochemical analyses were performed.

### Biochemical analysis

Salivary TAS and TOS levels were measured using commercially available colorimetric assay kits according to the manufacturers’ instructions. Salivary 8-OHdG and asprosin levels were analyzed using ELISA kits, while arylesterase (ARES) activity was measured using a commercial colorimetric assay kit. All saliva samples were analyzed in duplicate in accordance with the manufacturers’ instructions. The oxidative stress index (OSI) was calculated by dividing the TOS values by the TAS values. Detailed information regarding the assay kits, including the manufacturer names, catalog numbers, detection ranges, measurement units, assay sensitivity, and intra-/inter-assay coefficients of variation, is presented in Table 1.

**Table 1:** Characteristics of the assay kits used in the biochemical analyses

Biomarker	Method	Manufacturer	Catalog No.	Detection Range	Measurement Unit	Sensitivity	Intra-assay CV	Inter-assay CV
Asprosin	ELISA	BT-Lab	E4095Hu	0.5–100 ng/mL	ng/mL	0.23 ng/mL	< 8%	< 10%
8-OHdG	ELISA	Elabscience	E-EL-0028	1.56–100 ng/mL	ng/mL	0.94 ng/mL	6.1–7.0%	6.3–8.8%
ARES	Colorimetric	Rel Assay Diagnostics	RL0055	12.5–750 U/L	U/L	N/A	2.8%	3.3%
TAS	Colorimetric	Rel Assay Diagnostics	RL0017	1.20–1.50 mmol/L	µmol Trolox equivalent/L	N/A	2.8%	3.3%
TOS	Colorimetric	Rel Assay Diagnostics	RL0024	4.00–6.00 µmol H₂O₂/L	µmol H₂O₂ equivalent/L	N/A	3.2%	3.9%

*N/A* Not applicable, sensitivity values were not reported by the manufacturer for the colorimetric assays

### Statistical analysis

Statistical analyses were performed using IBM SPSS Statistics software (version 25.0; IBM Corp., Armonk, NY, USA). The normal distribution of the data was evaluated using the Shapiro–Wilk test. Continuous variables are expressed as mean ± standard deviation or median (minimum–maximum) according to the data distribution. Comparisons among the three groups were performed using one-way analysis of variance (ANOVA) or Kruskal–Wallis tests, where appropriate. Post-hoc analyses were conducted using Tukey or Dunn tests. Correlations between biochemical parameters and dmft scores were evaluated using Spearman’s correlation analysis. A *p*-value of < 0.05 was considered statistically significant.

## Results

A total of 90 children were included in the study (control: *n* = 29; ECC: *n* = 30; S-ECC: *n* = 31). No statistically significant differences were observed among the groups regarding age (*p* = 0.20) or sex distribution (*p* = 0.69). Caries experience, evaluated using the dmfs and dfs indices, was lowest in the control group, moderate in the ECC group, and highest in the S-ECC group (Table 2).

**Table 2:** Demographic characteristics of the groups

	Control (*n* = 29)	ECC(*n* = 30)	S-ECC (*n* = 31)	*p*-value
Age (years)	4,9 ± 0,9	5,2 ± 0,7	5,1 ± 0,8	0,20^A^
Sex				0,69^B^
Female	12 (%41,4)	13 (%43,3)	16 (%51,6)	
Male	17 (%58,6)	17 (%56,7)	15 (%48,4)	
dmft	0 (0–0)	4 (1–5)	8 (5–16)	n/a
dmfs	0 (0–0)	7 (2–10)	15 (6–34)	n/a

Descriptive statistics are presented as * mean ± standard deviation or ** median (minimum–maximum). A One-Way Analysis of Variance (One-Way ANOVA), B Pearson’s χ2 test. n/a: No evaluation was performed

*ECC* Early childhood caries, *S-ECC* Severe early childhood caries

### Biomarker levels among the groups

No statistically significant differences were observed among the groups regarding asprosin (*p* = 0.901), TOS (*p* = 0.865), OSI (*p* = 0.422), or arylesterase levels (*p* = 0.471) (Table 3).

**Table 3:** Comparisons of the investigated biomarkers among the groups

	Control(*n* = 29)	ECC (*n* = 30)	S-ECC (*n* = 31)	*p*-value
Asprosin *	37,9 (29,2–44,0)	36,2 (29,4–55,2)	38,2 (29,4–53,0)	0,901^A^
TAS *	0,69 (0,51–0,80)^a^	0,50 (0,38–0,68) ^b^	0,68 (0,57–0,85)^a^	**0**,**014**^A^
TOS *	3,1 (0,9–7,3)	2,8 (0,8–8,2)	3,3 (2,0–6,8)	0,865^A^
OSI *	0,50 (0,13–1,10)	0,69 (0,23–1,37)	0,41 (0,33–0,75)	0,422^A^
Arylesterase**	1008,9 ± 220,8	1051,7 ± 238,1	981,8 ± 197,7	0,471^B^
8-OhdG **	79,6 ± 20,3^a^	88,1 ± 18,4^a^	104,3 ± 40,6^b^	**0**,**013**^C^

Descriptive statistics are presented as * median (25th percentile–75th percentile) or ** mean ± standard deviation. A Kruskal–Wallis test, B One-Way Analysis of Variance (One-Way ANOVA), C Welch One-Way Analysis of Variance (Welch ANOVA). No statistically significant difference was found between the groups indicated with the same lowercase letters (*p* > 0.05)

*ECC* Early childhood caries, *S-ECC* Severe early childhood caries

**Fig. 1:**
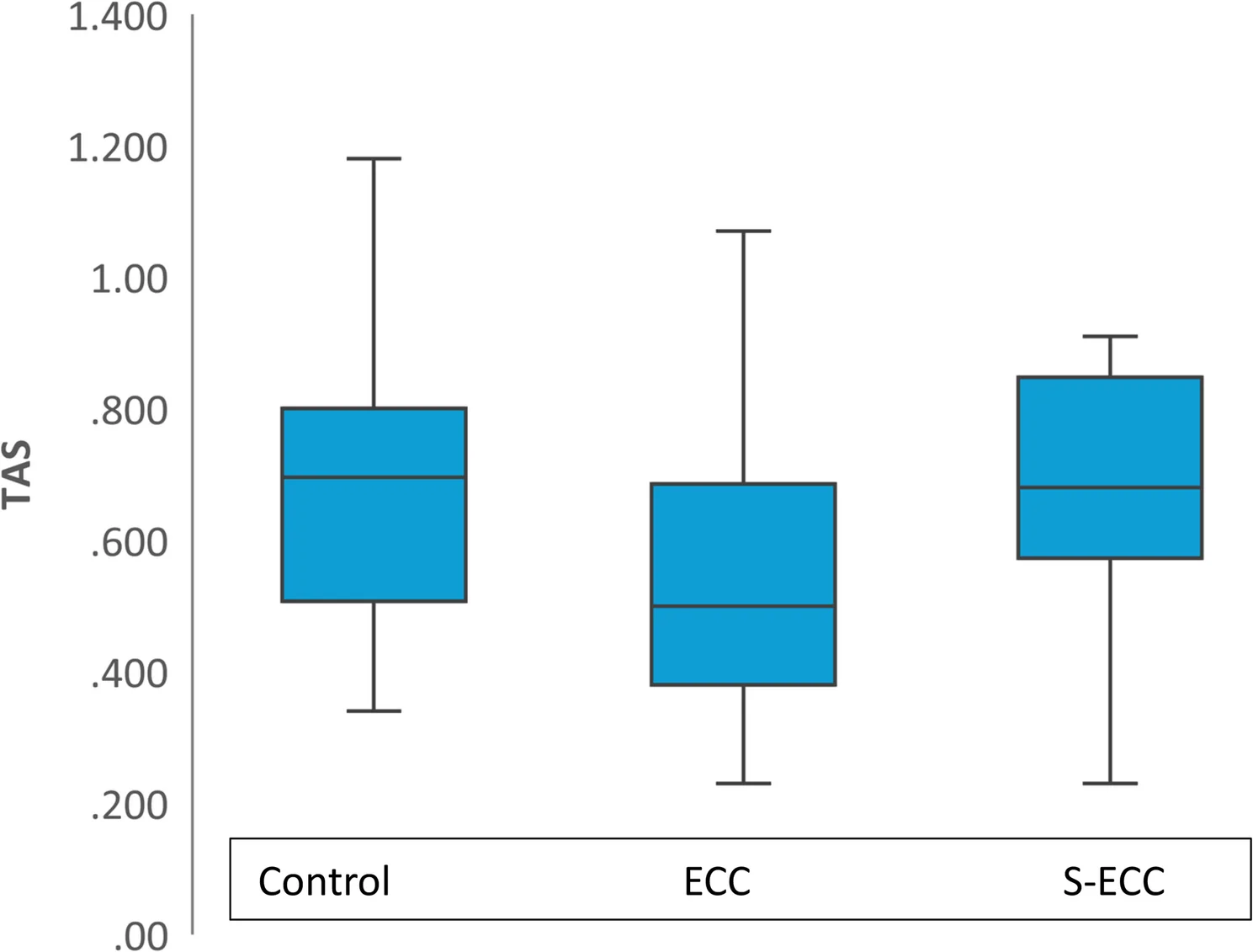
Comparison of TAS levels among the Control, ECC, and S-ECC groups

TAS levels were significantly different among the groups (*p* = 0.014). Post-hoc analyses revealed that TAS levels were significantly lower in the ECC group compared with both the control and S-ECC groups (respectively *p* = 0.041 and *p* = 0.033) (Table 3, Fig. 1).

**Fig. 2:**
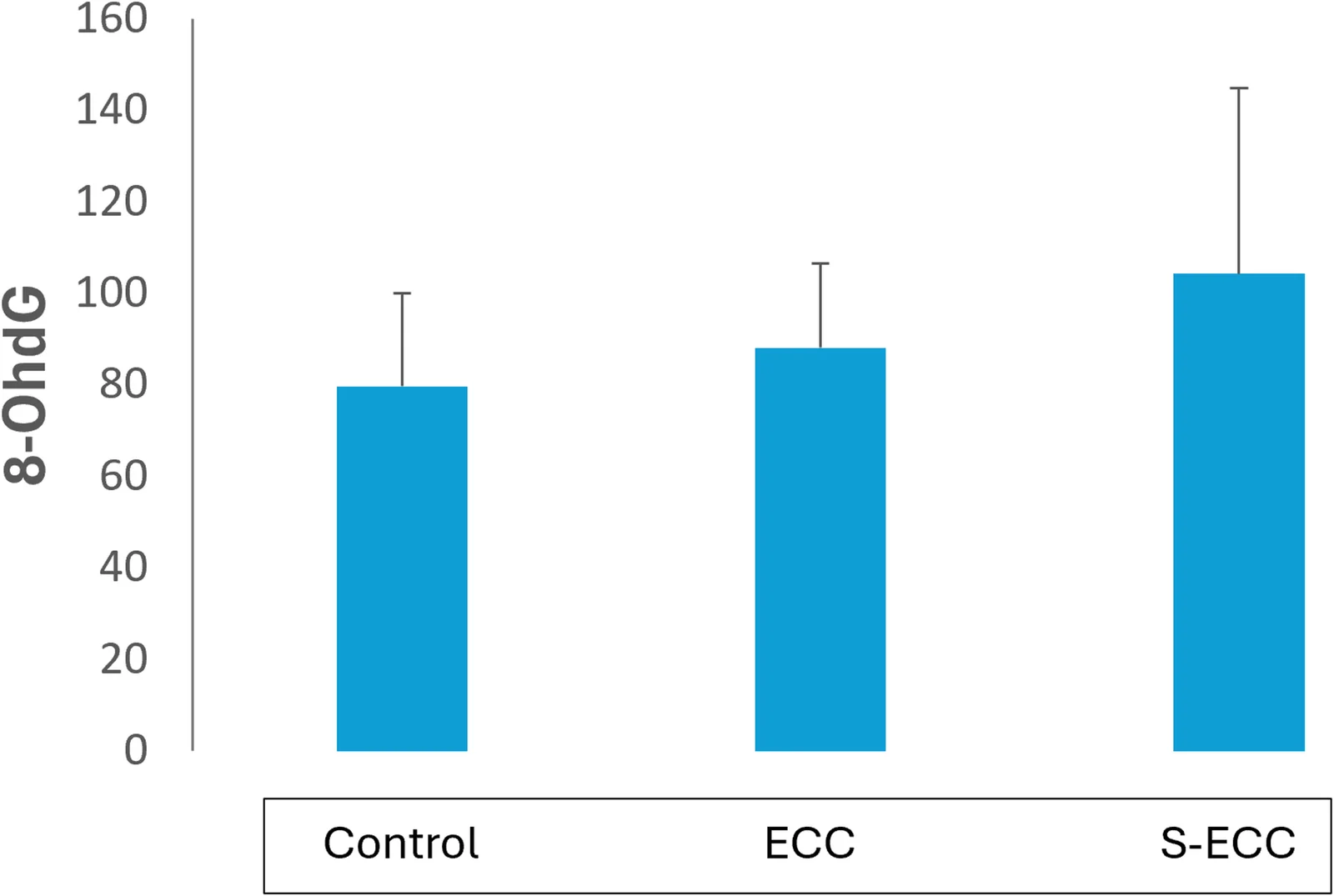
Graph showing the distribution of 8-OHdG levels according to the groups

Welch variance analysis demonstrated a significant difference among the groups in 8-OHdG levels (*p* = 0.013). Games–Howell post-hoc analysis revealed that 8-OHdG levels were significantly higher in the S-ECC group than in the control group (*p* = 0.012) (Table 3, Fig. 2).

### Correlation analysis

Spearman’s correlation analysis revealed a significant weak negative correlation only between 8-OHdG levels and age (*r* = − 0.253; *p* = 0.016). No significant correlations were found between the other biomarkers and age, dmft, or dfs scores (*p* > 0.05) (Table 4).

**Table 4:** Correlation coefficients and significance levels between age, dmft, dfs, and the investigated biomarkers among all cases

	Age	dmft	dfs
Asprosin			
Correlation Coefficient	-0,191	0,027	0,051
*p*-value^A^	0,07	0,80	0,63
TAS			
Correlation Coefficient	0,008	-0,008	-0,002
*p*-value ^A^	0,94	0,94	0,98
TOS			
Correlation Coefficient	-0,051	0,080	0,091
*P*-value ^A^	0,64	0,46	0,40
OSI			
Correlation Coefficient	-0,053	0,041	0,048
*p*-value ^A^	0,62	0,70	0,66
ARES			
Correlation Coefficient	0,026	-0,112	-0,127
*p*-value ^A^	0,81	0,30	0,24
8-OhdG			
Correlation Coefficient	-0,253	0,138	0,167
*p*-value ^A^	**0**,**016**	0,19	0,11

^A^ Spearman’s correlation analysis

Bold values indicate statistical significance (*p* < 0.05)

### Multiple linear regression analysis

Multiple linear regression analysis was performed to evaluate the effects of age and sex on the biomarker levels. The analysis demonstrated that age had a statistically significant negative effect on 8-OHdG levels (β = −0.253, *p* = 0.016). No significant differences were observed between females and males regarding asprosin (*p* = 0.370), TAS (*p* = 0.660), TOS (*p* = 0.430), OSI (*p* = 0.500), arylesterase (*p* = 0.110), or 8-OHdG levels (*p* = 0.620) (Table 5).

**Table 5:** Comparisons of the investigated biomarkers between females and males among all cases

	Female (*n* = 41)	Male (*n* = 49)	*p*-value
Asprosin *	33,6 (24,5–55,4)	37,9 (32,4–45,6)	0,37^A^
TAS *	0,57 (0,45–0,81)	0,66 (0,49–0,79)	0,66^A^
TOS *	3,1 (1,7–8,7)	3,2 (1,0–7,3)	0,43^A^
OSI *	0,56 (0,30–1,13)	0,45 (0,21–1,02)	0,50^A^
Arylesterase **	980,7 ± 213,9	1056,4 ± 220,9	0,11^B^
8-OhdG **	92,4 ± 22,6	89,2 ± 37,2	0,62^B^

Descriptive statistics are presented as * median (25th percentile–75th percentile) or ** mean ± standard deviation. A Mann–Whitney U test, B Student’s t-test

*ECC* Early childhood caries, *S-ECC* Severe early childhood caries

### Relationships among biomarkers

A moderate positive correlation was observed between TAS and TOS levels (*r* = 0.413; *p* < 0.001). A strong positive correlation was detected between TOS and OSI levels (*r* = 0.897; *p* < 0.001). Additionally, a weak negative correlation was identified between arylesterase and 8-OHdG levels (*r* = − 0.248; *p* = 0.020). No significant association was found between asprosin levels and other biomarkers (Table 6).

**Table 6:** Correlation coefficients and significance levels among the investigated biomarkers in all cases

	TAS	TOS	OSI	ARES	8-OhdG
Asprosin					
Correlation Coefficient	0,046	0,053	0,028	-0,111	0,054
*p*-value^A^	0,675	0,628	0,800	0,308	0,619
TAS					
Correlation Coefficient		0,413	0,066	-0,045	-0,010
*p*-value^A^		**< 0**,**001**	0,554	0,683	0,926
TOS					
Correlation Coefficient			0,897	0,020	0,120
*p*-value^A^			**< 0**,**001**	0,858	0,273
OSİ					
Correlation Coefficient				0,043	0,121
*p*-value^A^				0,695	0,270
ARES					
Correlation Coefficient					-0,248
*p*-value^A^					**0**,**020**

^A^ Spearman’s correlation analysis

Bold values indicate statistical significance (*p* < 0.05)

### Multivariable regression analysis

After adjustment for age and sex, being in the ECC group was associated with a significant decrease in TAS levels compared with the control group (B = − 0.250; 95% CI: −0.441 to − 0.058; *p* = 0.011).

Being in the S-ECC group was associated with a significant increase in 8-OHdG levels (B = 0.298; 95% CI: 0.104–0.491; *p* = 0.003).

No independent effects were identified for asprosin, TOS, OSI, or arylesterase levels (*p* > 0.05) (Table 7).

**Table 7:** Effects of early childhood caries and severe early childhood caries on changes in the investigated biomarkers after adjustment for age and sex

	B (%95 CI)	t-statistic	*p*-value
Asprosin			
Age	-0,095 (-0,219–0,029)	-1,527	0,131
Female Factor	-0,120 (-0,323–0,083)	-1,174	0,244
ECC	-0,020 (-0,268–0,227)	-0,163	0,871
S-ECC	0,071 (-0,179–0,320)	0,563	0,575
TAS			
Age	0,025 (-0,070–0,120)	0,53	0,598
Female Factor	-0,022 (-0,179–0,136)	-0,272	0,786
ECC	-0,250 (-0,441 – -0,058)	-2,594	**0**,**011**
S-ECC	-0,023 (-0,216–0,171)	-0,234	0,816
TOS			
Age	-0,063 (-0,420–0,293)	-0,354	0,724
Female Factor	0,251 (-0,338–0,841)	0,849	0,398
ECC	0,172 (-0,546–0,890)	0,477	0,635
S-ECC	0,313 (-0,412–1,037)	0,858	0,393
OSI			
Age	-0,079 (-0,416–0,258)	-0,468	0,641
Female Factor	0,202 (-0,357–0,762)	0,719	0,474
ECC	0,503 (-0,183–1,189)	1,46	0,148
S-ECC	0,230 (-0,454–0,915)	0,669	0,505
ARES			
Age	9,486 (-48,219–67,190)	0,327	0,744
Female Factor	78,117 (-16,710–172,944)	1,639	0,105
ECC	36,115 (-80,126–152,356)	0,618	0,538
S-ECC	-39,128 (-155,597–77,340)	-0,668	0,506
8-OhdG			
Age	-0,031 (-0,128–0,067)	-0,626	0,533
Female Factor	-0,136 (-0,294–0,022)	-1,707	0,091
ECC	0,137 (-0,058–0,333)	1,395	0,167
S-ECC	0,298 (0,104–0,491)	3,061	**0**,**003**

*B* Regression coefficient, *CI* Confidence interval

Bold values indicate statistical significance (*p *< 0.05)

## Discussion

This study comprehensively evaluated the effects of ECC on oxidative stress and the antioxidant defense system. The findings revealed that TAS levels were decreased in the ECC group, whereas 8-OHdG levels, indicating oxidative DNA damage, were increased in the S-ECC group. These findings suggest that oxidative stress may play a role in the pathogenesis and progression of dental caries.

Oxidative stress is known to be effective in the initiation and progression of many dental diseases, especially dental caries, which are one of the most common chronic diseases worldwide. TAS is a biomarker used to evaluate the total antioxidant defense level and measures the total radical-scavenging capacity of a biological sample [15].

The significantly lower TAS levels observed in the ECC group indicate that the antioxidant defense system is insufficient against the increased oxidative burden. Studies conducted in individuals with inflammatory conditions have reported increased TAS levels as a compensatory response [16–19], whereas studies reporting decreased TAS levels have suggested that antioxidant reserves are depleted due to excessive free radical production associated with inflammation [20, 21]. An increase in TAS has also been explained as a compensatory response to inflammation [22, 23]. Studies reporting decreased TAS levels have indicated that radical production exceeds antioxidant reserves [20, 24]. The decrease in TAS observed in our study may be explained by increased bacterial load and the resulting production of reactive oxygen species, leading to insufficient antioxidant defense mechanisms.

In our study, TOS levels did not differ significantly among the ECC, S-ECC, and control groups.This finding suggests that the oxidant load in saliva is not markedly affected by the presence of dental caries. Studies investigating the relationship between dental caries and salivary TOS levels are limited; however, some studies have reported increased TOS levels in individuals with dental caries [25]. However, no significant increase was observed in the present study. This finding may be explained by the fact that the oxidative response during the early stages of the inflammatory process may not have reached measurable levels in the saliva.

OSI, obtained from the ratio of TOS to TAS, is a parameter reflecting the oxidant–antioxidant balance as a whole [26]. Nevertheless, no significant differences were observed among the groups regarding the OSI values in our study. Although some studies have reported increased OSI levels in individuals with dental caries [27, 28], our findings do not support these results. Similarly, previous studies evaluating TAS and TOS in pediatric caries populations have yielded conflicting findings, with some authors reporting increased TAS, TOS, and OSI levels as a compensatory response to oxidative stress, whereas others have observed reduced antioxidant capacity associated with depletion of antioxidant reserves under conditions of persistent oxidative challenge [27, 28]. The inconsistent findings reported in previous studies regarding TAS, TOS, and OSI levels may be attributable to differences in study populations, age groups, caries severity, dentition type, saliva collection protocols, analytical methods, and sample sizes. In addition, variations in dietary habits and other environmental factors may contribute to the heterogeneity of the reported results. Therefore, the findings of the present study should be interpreted in the context of these methodological differences.

Despite decreased TAS levels in the ECC group, the absence of changes in the OSI may suggest that the oxidant–antioxidant balance was preserved on a proportional basis. This may be explained by the lack of significant changes in TOS levels, which limited the effect of the decrease in TAS on OSI. In addition, the biological variation in TOS and the limited oxidative variability specific to early childhood may have reduced the sensitivity of the OSI to caries severity.

The most remarkable finding of our study was the significantly increased 8-OHdG levels in the S-ECC group. To the best of our knowledge, there is no study in the literature investigating the relationship between ECC and 8-OHdG. However, increased 8-OHdG levels have been widely reported in periodontal diseases. The increase in periodontal diseases reflects oxidative DNA damage associated with inflammation and increased ROS production [29–31].

The absence of a significant increase in the ECC group suggests that oxidative DNA damage may occur after reaching a certain threshold of disease severity. This finding may indicate that severe early childhood caries is associated with increased oxidative DNA damage beyond the oral environment. Consistent with this observation, multivariate regression analysis demonstrated that the S-ECC group was independently associated with higher 8-OHdG levels. Another common finding in many studies and meta-analyses is that 8-OHdG levels, which are an important indicator of oxidative DNA damage, increase with age [32, 33]. In our study, a weak but statistically significant negative relationship was found between 8-OHdG levels and age (*r*=-0.253; *p* = 0.016). Since our study included only early childhood and had a narrow age range, the absence of the age-related increase trend reported in the literature in our samples is an expected finding.

Asprosin is a peptide-structured hormone secreted from white adipose tissue and has important functions in the regulation of glucose homeostasis and energy metabolism [34]. In this study, asprosin levels did not differ significantly among the groups. Previous studies have reported that asprosin is associated with metabolic processes and systemic inflammation [35, 36]. Therefore, the absence of significant changes in dental caries, which are localized infections, may be an expected finding. Similarly, the absence of significant differences in arylesterase levels may suggest that oxidative stress does not markedly affect the enzymatic antioxidant system in asymptomatic cases.

This study had several limitations. First, the sample included only the early childhood period and had a narrow age range, which limited the evaluation of age-related changes in biomarkers. Second, although saliva samples were collected under standardized conditions, oral hygiene status, dietary habits, sugar consumption, body mass index (BMI), and socioeconomic status were not specifically assessed. These factors may influence oxidative stress biomarkers and, therefore, may have affected the observed biomarker levels in the present study. Accordingly, these factors should be considered potential confounding factors when interpreting the findings. Future studies incorporating these variables may provide a more comprehensive understanding of the relationship between early childhood caries and oxidative stress.

## Conclusion

In this study, biomarkers related to oxidative stress and the antioxidant defense system in saliva were comprehensively evaluated in children with ECC and S-ECC.

The findings obtained demonstrated that TAS significantly decreased in the ECC group, whereas oxidative DNA damage markers and 8-OHdG levels significantly increased in the S-ECC group. In contrast, no significant differences were found among the groups regarding asprosin, TOS, OSI, and arylesterase levels.

As a result, the decrease in antioxidant defense in ECC and the increase in oxidative DNA damage in S-ECC indicate that the caries process may be associated with oxidative mechanisms. This situation emphasizes the importance of approaches supporting antioxidant defense during the early period.

## Data Availability

All data generated or analyzed during this study are included in this published article.

## Abbreviations

ARES: Arylesterase
BMI: Body mass index
CI: Confidence interval
CV: Coefficient of variation
ECC: Early childhood caries
ELISA: Enzyme-linked immunosorbent assay
OSI: Oxidative stress index
ROS: Reactive oxygen species
S-ECC: Severe early childhood caries
TAS: Total antioxidant status
TOS: Total oxidant status
8-OHdG: 8-Hydroxy-2′-deoxyguanosine
